# Pituitary transcriptome profile from laying period to incubation period of Changshun green-shell laying hens

**DOI:** 10.1186/s12864-024-10233-1

**Published:** 2024-03-25

**Authors:** Zhi Chen, Di Wen, Yan Zhang, Jiaying Chen, Fengqian Pan, Wen Zhang, Shuangshuang Zhou, Fen Wang, Ren Mu

**Affiliations:** 1 College of Biological Science and Agriculture, Qiannan Normal University for Nationalities, 558000 Duyun, China; 2 Qiannan Key Laboratory of Applied Biotechnology for Livestock and Poultry, 558000 Duyun, China

**Keywords:** Changshun green-shell laying hens, Pituitary, Transcriptome analysis, Laying period, Incubation period

## Abstract

**Background:**

Incubation behaviour, an instinct for natural breeding in poultry, is strictly controlled by the central nervous system and multiple neuroendocrine hormones and neurotransmitters, and is closely associated with the cessation of egg laying. Therefore, it is essential for the commercial poultry industry to clarify the molecular regulation mechanism of incubation behaviour. Here, we used high-throughput sequencing technology to examine the pituitary transcriptome of Changshun green-shell laying hen, a local breed from Guizhou province, China, with strong broodiness, in two reproductive stages, including egg-laying phase (LP) and incubation phase (BP). We also analyze the differences in gene expression during the transition from egg-laying to incubation, and identify critical pathways and candidate genes involved in controlling the incubation behaviour in the pituitary.

**Results:**

In this study, we demonstrated that a total of 2089 differently expressed genes (DEGs) were identified in the pituitary, including 842 up-regulated and 1247 down-regulated genes. Kyoto Encyclopedia of Genes and Genomes (KEGG) analysis revealed that steroid biosynthesis pathway and neuroactive ligand-receptor interaction were significantly enriched based on DEGs commonly identified in pituitary. Further analysis revealed that SRC, ITGB4, ITGB3, PIK3R3 and DRD2 may play crucial roles in the regulation of incubation behaviour.

**Conclusions:**

We identified 2089 DEGs and the key signaling pathways which may be closely correlated with incubation in Changshun green-shell laying hens, and clarified the molecular regulation mechanism of incubation behaviour. Our results indicate the complexity and variety of differences in reproductive behaviour of different chicken breeds.

**Supplementary Information:**

The online version contains supplementary material available at 10.1186/s12864-024-10233-1.

## Introduction

Incubation behaviour, also colloquially known as broodiness, is a behaviour commonly occurring in domestic poultry. In domestic chickens, incubation behaviour consists of persistent nesting, turning and retrieval of eggs, characteristic clucking, and defense of the nest [1, 2]. It can cause atrophy of the fallopian tubes and ovaries, and the subsequent cessation of egg laying, leading to economic losses for the domestic poultry industry [1]. Although the mechanism of incubation behaviour has been extensively investigated in domestic chickens, the exact regulatory mechanisms remain elusive. Usually, incubation behaviour is known to be strictly controlled by the central nervous system and is closely associated with multiple neuroendocrine hormones and neurotransmitters.

During the transition from egg-laying to incubation in domestic chickens, the levels of hormones change markedly, and prolactin (PRL) is considered to be a main factor induced during incubation. The onset and maintenance of incubation behaviour in chickens are demonstrated to be closely correlated with a characteristic increase in plasma PRL level [3–7]. Meanwhile, the concentrations of luteinizing hormone (LH) and steroid hormones in plasma decrease dramatically during incubation, leading to regression of the ovary [5]. Once the young chicks have hatched, the plasma PRL level subsequently declines dramatically, and the concentrations of LH and estradiol increase gradually [5, 8–10]. Increased PRL and decreased LH secretion in incubating chickens are found to be correlated with increased hypothalamic neuropeptide vasoactive intestinal polypeptide (VIP) and decreased hypothalamic gonadotropin-releasing hormone (GnRH), respectively. The hypothalamic VIP is a physiological PRL-releasing hormone in chickens. It can not only increase the gene expression of *PRL* in pituitary gland, but also induce the release of PRL by acting directly on the the lactotrophs of pituitary gland [11–14]. In addition, the neurotransmitters are also thought to be involved in the regulation of avian incubation behaviour. The neurotransmitter dopamine (DA) has both an inhibitory and stimulatory effect on the secretion of avian PRL, depending on the subtype of DA receptor [15, 16]. However, 5-hydroxytryptophan (5-HTP) and dynorphin are reported to stimulate VIP and PRL release, respectively [17, 18].

In addition to neuroendocrine control, many investigations on the genetic control of incubation behaviour have been reported in the past decades. However, these investigations appear to have led to conflicting observations. Previous studies have extensively reported that incubation behaviour is a polygenic trait, and is controlled by more than one independent autosomal gene [19, 20]. While, subsequent investigations indicated that incubation behaviour might be controlled, at least to a large extent, by Z-linked genes, which seems to be contradictory to previous findings [21, 22]. Interestingly, the quantitative trait loci (QTL) analysis indicated that genetic loci affecting incubation behaviour are not located on the Z chromosome, but on chromosomes 1, 5, 8, 13, 18 and 19 and linkage group E22C19W28 [23]. Overall, it is now generally accepted that more than one locus controls the incubation behaviour in domestic chickens.

Changshun green-shell laying hen is an indigenous breed of chicken in Guizhou province, China. It is known to be a dual purpose egg- and meat-type chicken, and their eggs are considered to be superior in local markets for their better appearance, higher contents of protein, better amino acid composition, and lower fat and cholesterol content [24]. Although its strong tendency for broodiness significantly reduces egg production, and has restricted development of the Changshun chicken industry, the stable laying-incubation cycles and easily identifiable characteristics of broodiness make it an ideal model to study incubation behaviour and the molecular regulatory mechanism of poultry broodiness.

In recent years, RNA sequencing (RNA-Seq) has been widely used to investigate the dynamic gene expression profiles of key genes in the hypothalamic-pituitary-gonadal (HPG) axis of poultry between the laying period and incubation period [25–27]. A total of 110 differentially expressed genes (DEGs) were screened in the pituitary between laying and nesting geese, and the PRL, oxytocin-neurophysin (OXT), chordin-like protein 1 (CHRDL1) and growth hormone (GH), expressed in the pituitary gland, are considered as new candidate molecules involved in incubation in geese [25]. In ducks, 398 DEGs were identified in pituitary during the transition from egg-laying to incubation, and Gene Ontology (GO) and Kyoto Encyclopedia of Genes and Genomes (KEGG) analysis further indicated that neuroactive ligand-receptor interaction pathway, calcium signaling pathway, and response to steroid hormones biological process are critical for the transition from egg-laying to incubation phase of ducks [27]. Considering the complexity and variety of differences in reproductive behaviour of different poultry breeds, it is essential to understand the genetic basis of the transition from egg-laying to incubation phase of chickens at transcriptome level.

A large number of studies have focused on the identification of potential genes and gene regulatory networks associated with high rates of egg production in chicken pituitary [28, 29]. However, there are few studies on pituitary transcriptome analysis of incubation behaviour in chickens. Here, we systematically examined the pituitary transcriptome of Changshun green-shell laying hens at LP and BP through high-throughput sequencing and concentrated on the analysis of the differences in gene expression during the transition from LP to BP. Candidate genes involved in incubation were screened out, and related pathways were evaluated through KEGG enrichment and GO enrichment analysis. This study further reveals the complexity and variety of differences in reproductive behaviour of different chicken breeds and provide new insights into poultry reproductive behaviour.

## Materials and methods

### Ethics statement

The animal experiments were performed under the guidelines published in the Guide for the Care and Use of Laboratory Animals of the Ministry of Science and Technology of the People’s Republic of China. All methods strictly follow ARRIVE (Animal Research: Reporting of In Vivo Experiments) guidelines 2.0. The experimental procedures in this study were approved by the Animal Ethics Committee of the College of Biological Science and Agriculture, Qiannan Normal University for Nationalities, Duyun, China (AEC No. QNUN2020018).

### Animals and sample preparation

**Fig. 1:**
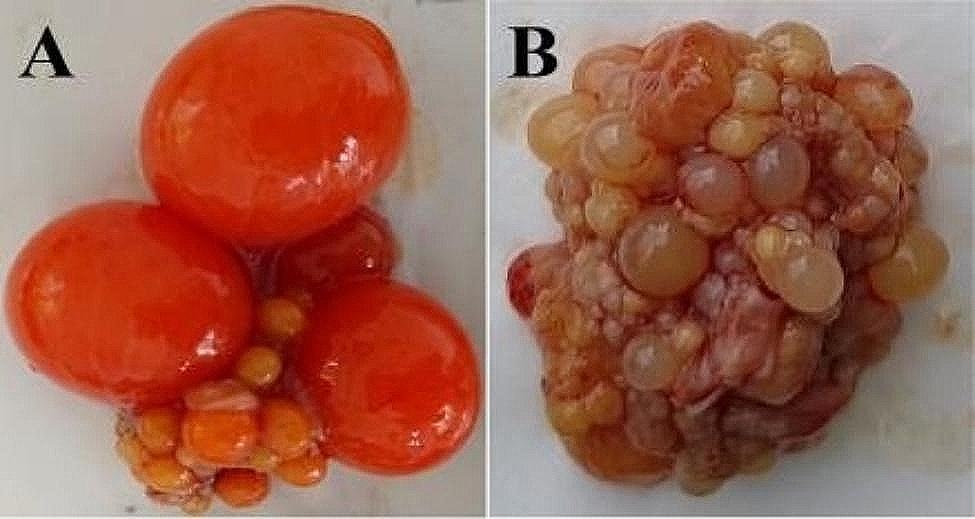
Ovary morphology of hens at LP (A) and BP (B)

The experimental Changshun green-shell laying hens were obtained from Changshun Sanyuan Agricultural Development Co., Ltd., China and were then raised in the poultry breeding farm of Qiannan Normal University for Nationalities according to the standard procedure. 100 females and 15 males were reared on the floor with litter and artificial nest and exposed to natural temperature conditions. An infrared video camera was set up above laying nest. The incubation behaviours were then recorded daily in the annual reproductive cycle. The individuals with high fertility were deemed to be LP birds. The hens which sat in the nest while exhibiting characteristic clucking, aggressive or defensive behaviour for at least one week were allocated as the BP group. Six hens from LP and BP stages were randomly chosen according to their behaviours and the ovary and anterior and posterior pituitary gland samples were quickly collected after euthanization by exsanguination under sodium pentobarbital anesthesia (60 mg/kg), respectively. The morphologic characteristics of the ovaries in the study were mainly used to further evaluate individual physiological stage of chickens (Fig. 1). All the anterior and posterior pituitary samples were immediately frozen in liquid nitrogen and then stored at -80 °C until total RNA extraction for transcriptome sequencing. Three pituitary samples from each of the LP and BP groups were randomly chosen for transcriptome sequencing according to the morphologic characteristics of the ovary.

### RNA extraction, cDNA library construction, and mRNA sequencing

Total RNA from three individuals in each of the LP and BP groups was extracted from the pituitary samples using the Trizol reagent (Life technologies, California, USA), according to the manufacturer’s instruction manual. The concentration and quality of the total RNA were determined using NanoDrop 2000 (Thermo Fisher Scientific, Wilmington, DE). RNA integrity was evaluated using the RNA Nano 6000 Assay Kit of the Agilent Bioanalyzer 2100 system (Agilent Technologies, CA, USA). Sequencing libraries were generated using Hieff NGS Ultima Dual-mode mRNA Library Prep Kit for Illumina (Yeasen Biotechnology (Shanghai) Co., Ltd.) following manufacturer’s recommendations. The libraries were sequenced on an Illumina NovaSeq6000 platform using 150 bp paired-end reads, according to the manufacturer’s instructions.

### Data analysis

The raw reads were further processed with a bioinformatics pipelinetool, BMKCloud (www.biocloud.net) online platform. Raw sequences were firstly transformed into clean reads through removing adaptor sequences and discarding low-quality sequences. Hisat2 tools software (v2.2.1, -dta -p 6 -max-intronlen 5,000,000) was then used to map against the chicken reference genome (Gallus_gallus.GRCg6a_release106.genome.fa) [30]. Both known and novel transcripts from Hisat2 alignment results were detected by the method of the StringTie Reference Annotation Based Transcript (v2.2.1, -merge -F 0.1 -T 0.1) [31]. The KEGG (http://www.genome.jp/kegg/), National Center for Biotechnology Information for non-redundant proteins (ftp://ftp.ncbi.nih.gov/blast/db/), Eukaryotic Orthologous Groups of proteins (http://www.ncbi.nlm.nih.gov/KOG/), Protein family (http://pfam.xfam.org/), GO (http://www.geneontology.org/) and Swiss-Prot (http://www.uniprot.org/) databases were used to annotate the gene function. The principal component analysis (PCA) was applied to assess the similarity between the samples.

### Identification of DEGs

Differential expression analysis of between the two groups was performed using DESeq2 (v1.30.1) [32]. DESeq2 provides statistical routines for determining differential expression in digital gene expression data using a model based on the negative binomial distribution. The resulting *P* values were adjusted using the Benjamini and Hochberg’s approach for controlling the false discovery rate. Genes with an adjusted *P*-value < 0.05 & Fold Change ≥ 1.5 found by DESeq2 were assigned as differentially expressed.

### KEGG pathway, GO enrichment analysis and protein-protein interaction

GO enrichment analysis of DEGs was implemented by the clusterProfiler package (v3.16.1) based Wallenius non-central hyper-geometric distribution, and KOBAS software (v3.0) was used to test the statistical enrichment of DEGs in KEGG pathways [33, 34]. The protein-protein interaction (PPI) analysis was performed using Cytoscape [35].

### Gene expression analysis by quantitative real-time PCR

Using total RNA, 1 µg was reverse transcribed to cDNA using the Prime Script RT reagent Kit (Takara, Dalian, China). Four genes of interest, including *PRL*, *BMP5*, *DHCR7* and *SC5D*, were selected to validate the RNA-seq results. *Beta-actin* was chosen as an internal reference gene for the normalization of expression levels. The primers used in the quantitative real-time PCR (qRT-PCR) were designed using Primer 5 (Table S1). Each assay was carried out in triplicate. The relative mRNA expression levels were calculated by the 2^−ΔΔCt^ method [36].

### Statistical analysis

Statistical analysis was performed using the R software (v4.0.2, R Development Core Team 2019). Data were analyzed using the Student’s t-test after testing for the homogeneity of variance with Levene’s test. All data are presented as the mean ± SD, and statistical significance is shown as *P < 0.05.

## Results

### RNA sequencing quality assessment

A total of six libraries were constructed from the pituitary and sequenced using Illumina NovaSeq platform. The reads of transcriptome data and their statistical analysis are shown in Table 1. The clean reads from each library averaged more than 19 million. The base percentage of the Q20 (base sequencing error probability < 1%) and Q30 (base sequencing error probability < 0.1%) ranged from 98.15 to 98.46% and 94.72–95.42%, respectively, indicating high accuracy of the sequencing. The GC content of all pituitary samples ranged from 47.98 to 49.54%. The above results showed that the sequencing data was suitable for subsequent data analysis.

**Table 1:** Quality metrics of transcripts in pituitary

Sample	Clean reads	Clean bases	Q20 (%)	Q30 (%)	GC content (%)	Total mapped	Multiple mapped	Uniquely mapped
LP1	21,699,331	6,489,415,358	98.20	94.77	49.15	20,291,871 (93.51%)	398,685 (1.84%)	19,893,187 (91.68%)
LP2	23,189,413	6,932,116,122	98.33	95.10	49.24	21,704,639 (93.60%)	461,979 (1.99%)	21,242,661 (91.60%)
LP3	19,290,472	5,767,577,132	98.15	94.72	49.54	17,998,306 (93.30%)	440,208 (2.28%)	17,558,098 (91.02%)
BP1	24,468,336	7,318,629,774	98.43	95.27	47.98	23,215,360 (94.88%)	741,255 (3.03%)	22,474,105 (91.85%)
BP2	20,122,438	6,020,221,454	98.46	95.42	48.47	18,942,509 (94.14%)	671,406 (3.34%)	18,271,104 (90.80%)
BP3	21,727,047	6,500,165,130	98.40	95.27	48.79	20,484,626 (94.28%)	650,102 (2.99%)	19,834,525 (91.29%)

Note: Q20 and Q30 represent error rate less than 1% and 0.1%, respectively. GC content presents the percentage of G and C bases in clean data

### Transcriptome alignment

As shown in Table 1, the mapping rate of the clean reads from six pituitary samples ranged from 93.30 to 94.88%. The percentage of multiple and uniquely mapped reads in clean reads ranged from 1.84 to 3.34% and 90.80–92.22%, respectively. The results indicated that the transcriptome data were reliable and suitable for subsequent analysis.

### Differentially expressed genes

**Fig. 2:**
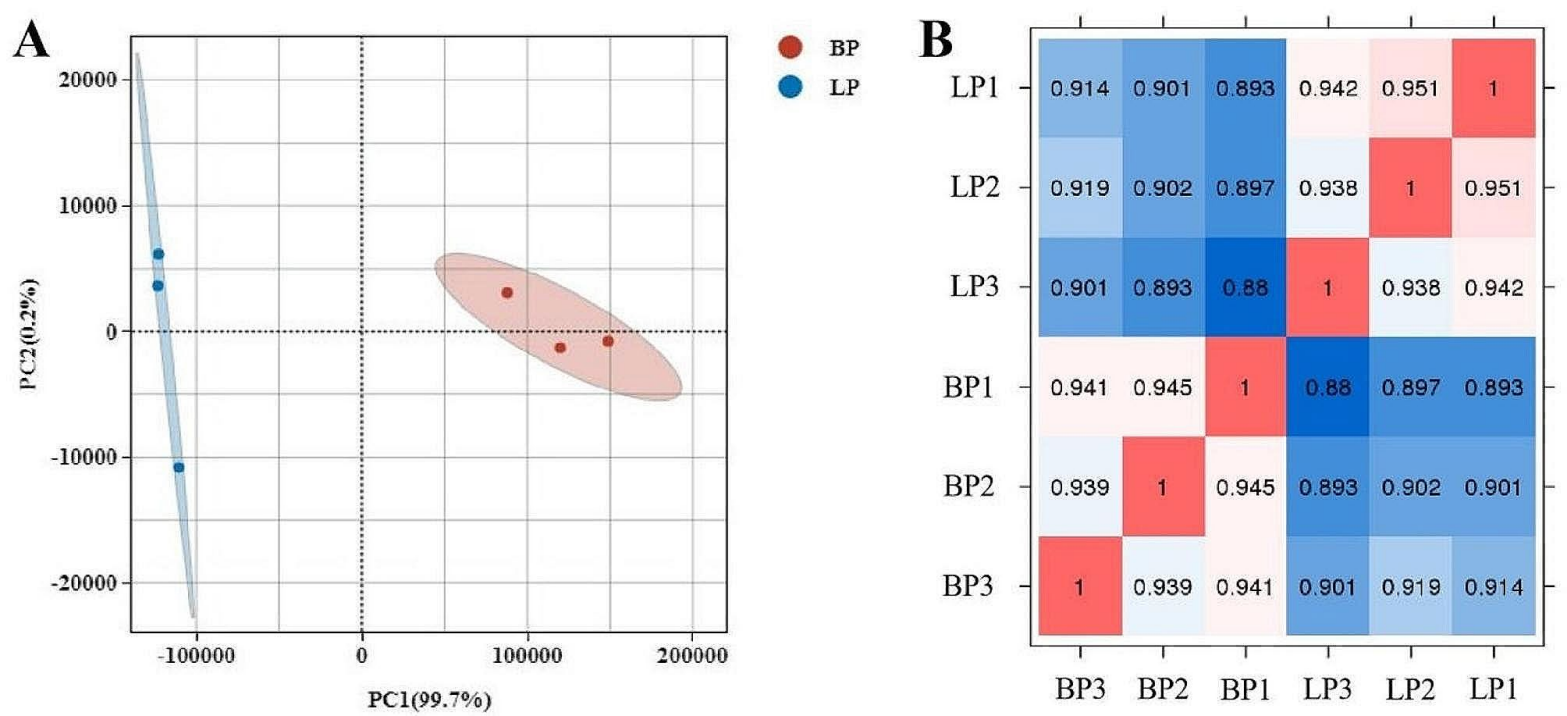
Features of sequencing data. (**A**) PCA score plot of pituitary transcriptomes. Blue and red nodes represent individuals from egg-laying phase (LP) and incubation phase (BP), respectively. (**B**) Pearson correlation analysis of LP and BP samples. LP1, LP2 and LP3 are pituitary samples of egg-laying phase hens, and BP1, BP2 and BP3 are pituitary samples of incubation metaphase hens

**Fig. 3:**
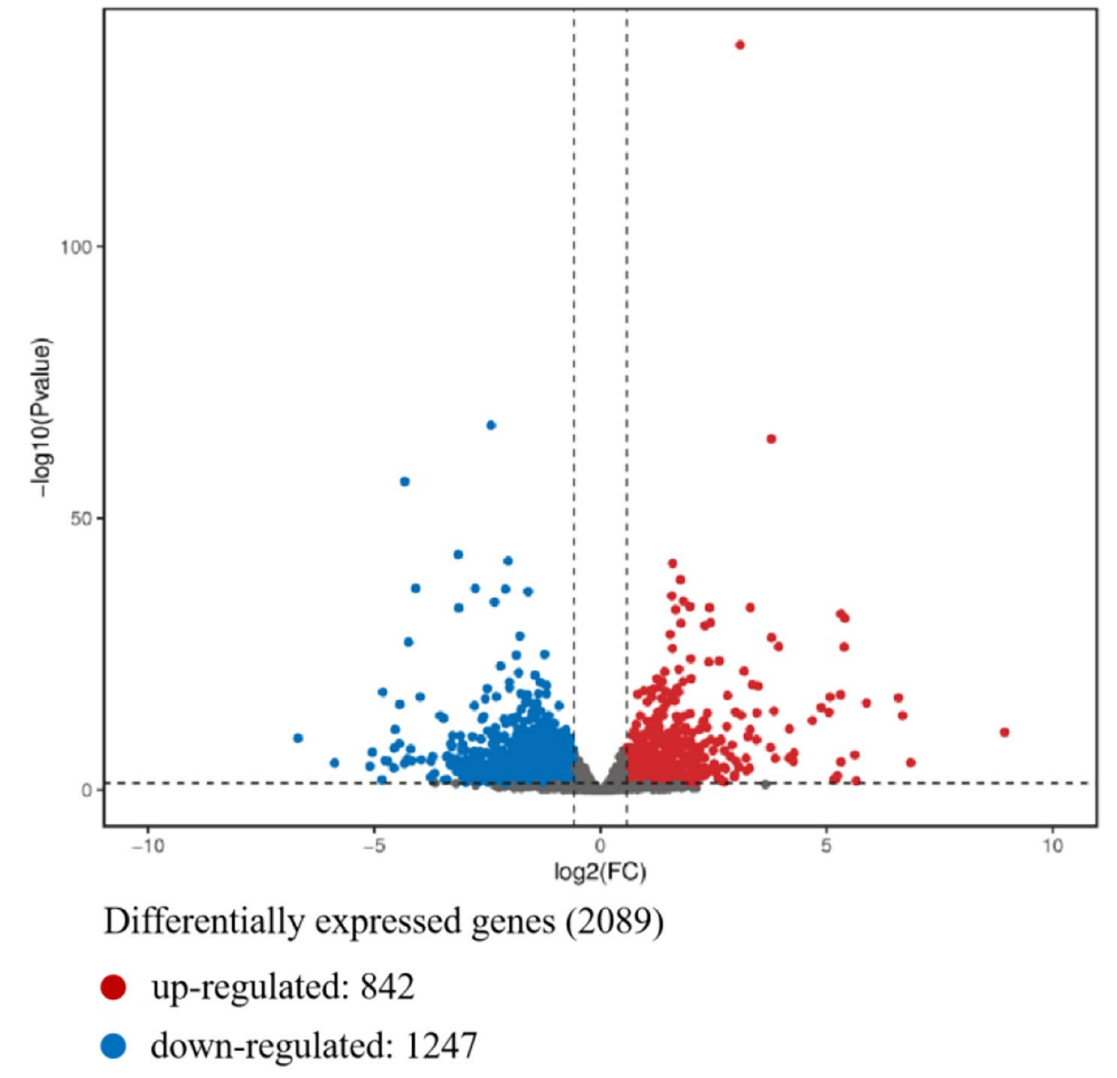
Volcano map of all expressed genes. The longitudinal and horizontal coordinates represent the statistical significance of the changes in gene expression and the fold changes of genes between the LP and BP groups, respectively. Each dot in the map represents one gene. Red and blue represent significantly up- and down-regulated genes, respectively. Gray represents genes showing no significant difference

Principal component analysis (PCA) was first conducted to analyze the six samples in LP and BP. As shown in Fig. 2A, the pituitary samples in LP and BP groups were separated from each other, indicating an obvious difference in mRNA expression between these two groups. The correlation coefficient of gene expression levels between samples demonstrated good sample repeatability in each group (Fig. 2B). A comparison of the gene expression showed that a total of 2089 DEGs were identified in the pituitary, including 842 up-regulated and 1247 down-regulated genes in the BP group (Fig. 3 and Table S2). Subsequently, hierarchical clustering analysis was carried out on the DEGs. We observed that the pituitary samples of LP and BP groups were distributed in different sub-clusters, but the three biological replicates from each group all clustered closely together. The heatmap visually reflected the expression patterns of the genes in the samples, indicating the reliability of screened DEGs (Figure S1).

### KEGG pathway and GO enrichment analysis

**Fig. 4:**
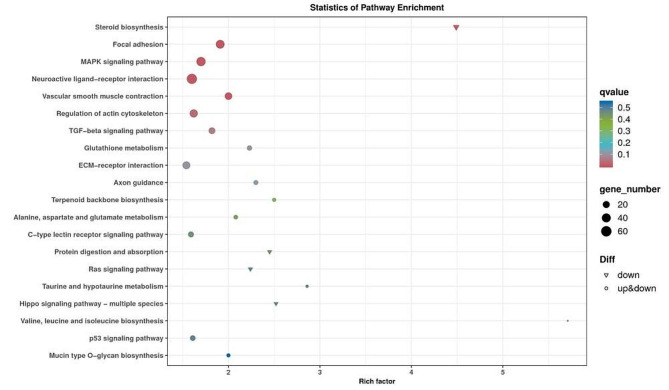
KEGG pathway enrichment analysis of DEGs in pituitary. Bubble charts represent the top 20 significantly enriched KEGG pathways (with smallest q value). Each circle represents a KEGG pathway. Size of each circle represents the number of DEGs in each pathway and the colour represents the q value of each pathway. The enrichment factor is the ratio of the number of DEGs enriched in the pathway compared to the total number of all annotated genes enriched in this pathway

**Fig. 5:**
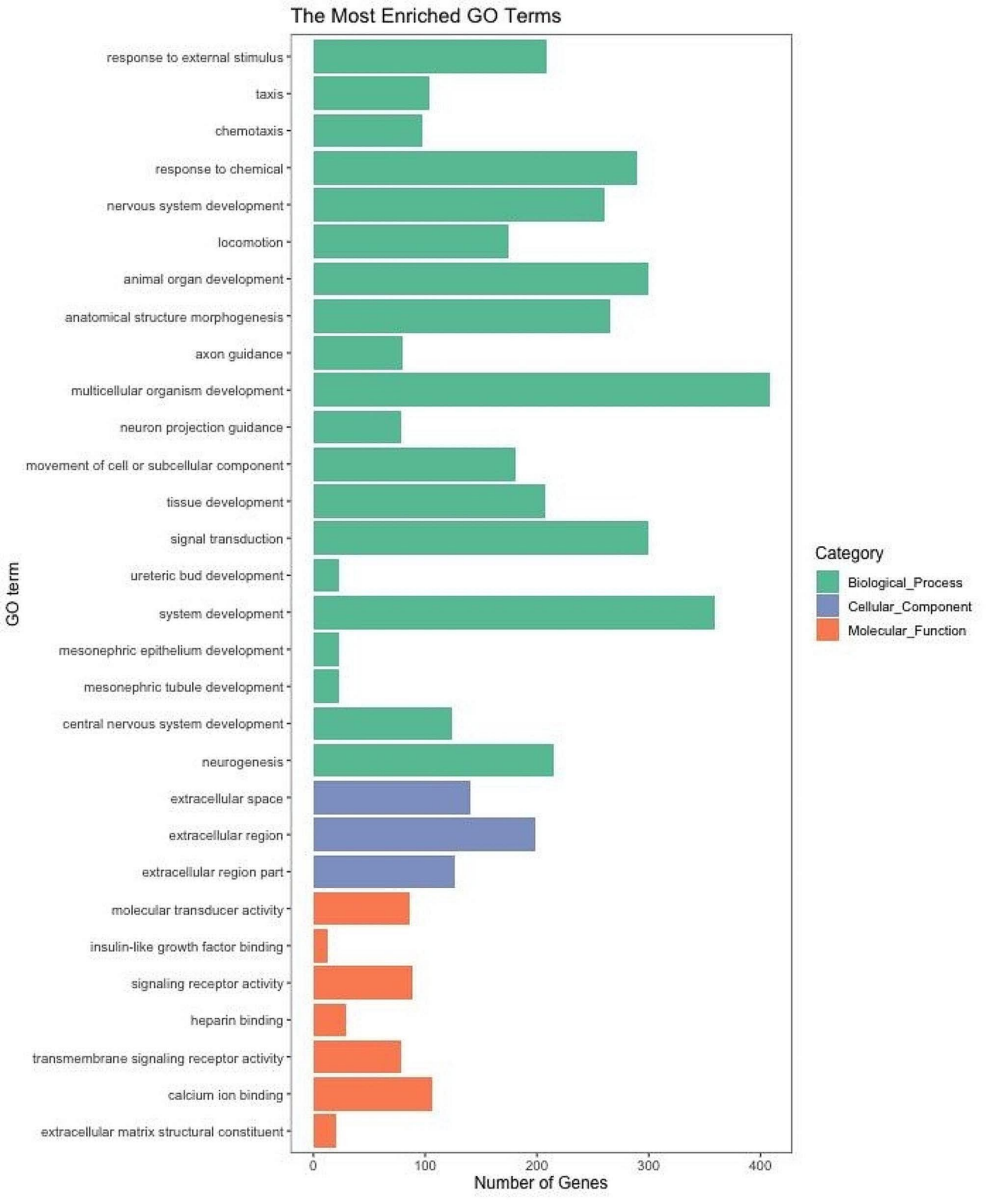
GO classification of DEGs identified in pituitary. The longitudinal coordinates represent the number of DEGs annotated to the term. The horizontal coordinates represent the GO terms and classifications

The KEGG pathway enrichment analysis was performed to further identify the major biochemical, metabolic, and signal transduction pathways of the DEGs. The top 20 significantly enriched KEGG pathways are listed in Fig. 4. The ten most enriched pathways were steroid biosynthesis, focal adhesion, MAPK signaling pathway, neuroactive ligand-receptor interaction, vascular smooth muscle contraction, regulation of actin cytoskeleton, TGF-beta signaling pathway, glutathione metabolism, ECM-receptor interaction and axon guidance. Notably, valine, leucine and isoleucine biosynthesis had the largest enrichment factor. Subsequently, the DEGs were annotated with Gene Ontology (GO) database into three main categories namely biological process (BP), cellular component (CC) and molecular function (MF) and 31 subcategories (Fig. 5 and Table S3). Based on the GO classification, 22 subcategories were assigned to the BP, 10 and 4 subcategories to the MF and CC, respectively (Table S3).

### Interaction network construction of DEGs

**Fig. 6:**
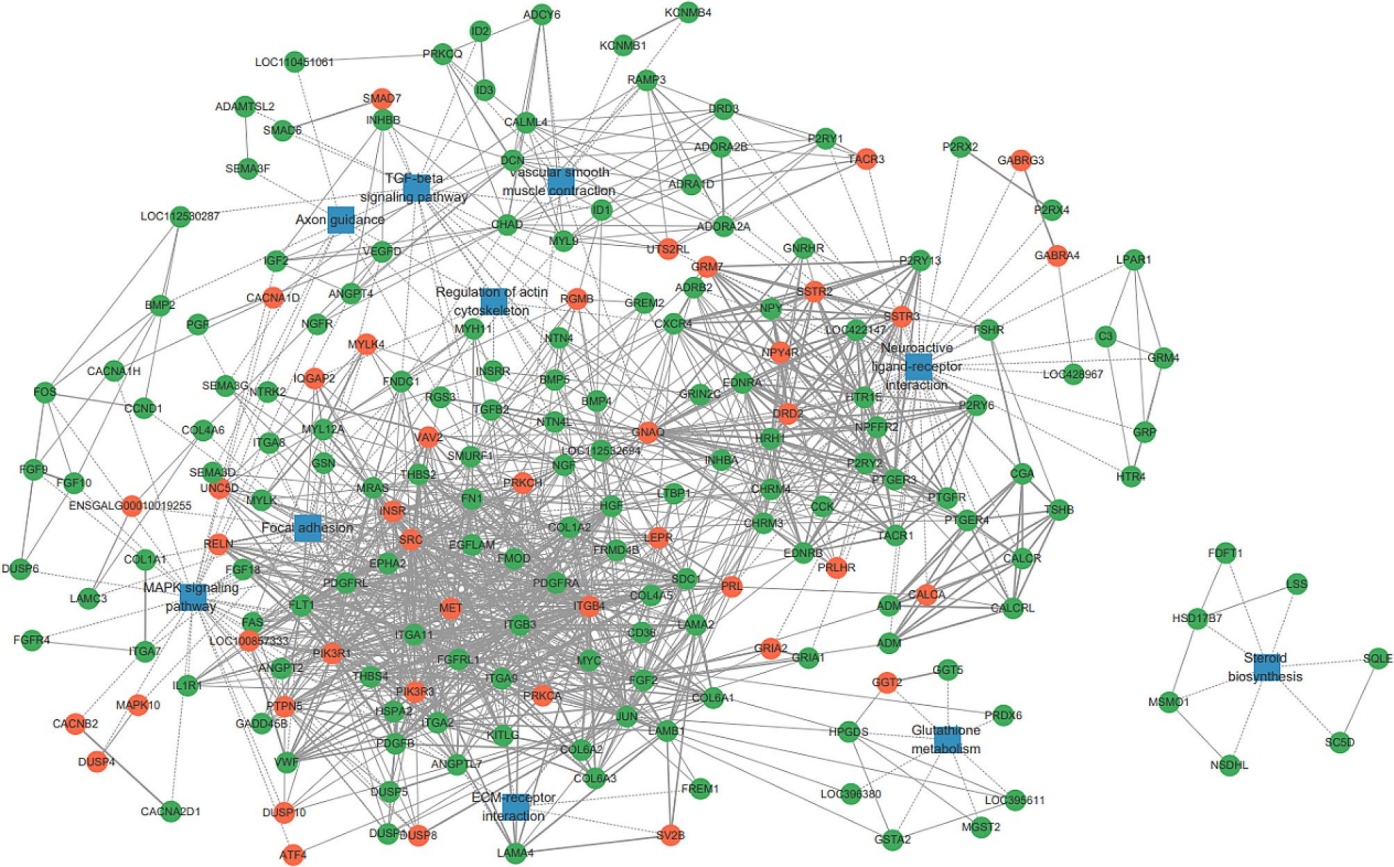
PPI networks of DEGs in pituitary. Circle nodes for genes/proteins, rectangle nodes for KEGG pathway. In case of fold change analysis, genes/proteins were coloured in red (upregulation) and green (downregulation). Interactions are shown as solid lines between genes/proteins, and edges of KEGG pathway in dashed lines

To further explore the biological characteristics of these DEGs from the pituitary, an analysis of protein-protein interaction (PPI) networks was performed (Fig. 6). The PPI networks were visualized by Cytoscape based on information gained up to 3 levels of functional analysis: fold change of genes, PPI and KEGG pathway. The PPI network of DEGs consisted of 209 nodes and 1252 edges. Functional analysis further indicated that the PPI network was mainly enriched into 10 important pathways including steroid biosynthesis, neuroactive ligand-receptor interaction, MAPK signaling pathway, ECM-receptor interaction, glutathione metabolism, focal adhesion, regulation of actin cytoskeleton, vascular smooth muscle contraction, axon guidance and TGF-beta signaling pathway. The top five genes with the highest degree in the PPI network were *SRC*, *ITGB4*, *ITGB3*, *PIK3R3* and *DRD2*, which may play important roles in mediating the transition from egg-laying to incubation in chickens.

### Verification of DEGs by qRT-PCR

**Fig. 7:**
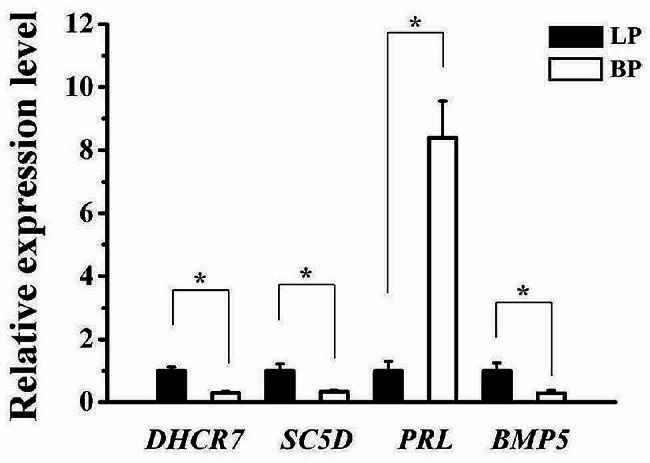
qRT-PCR validation of DEGs. The relative mRNA expression levels were calculated by the 2^−ΔΔCt^ method. The results were expressed as mean ± SD. * for *p* < 0.05

To validate the RNA-seq results, four DEGs identified in pituitary, including *PRL*, *BMP5*, *DHCR7* and *SC5D*, were selected for qRT-PCR analysis. While there are clear differences in the expression levels of these four genes, the expression trends determined by the qRT-PCR were consistent with the RNA-Seq results, indicating that the RNA-seq results were reliable (Fig. 7).

## Discussion

Broodiness is known to be a complicated maternal behaviour which consists of cessation of egg laying, the incubation of eggs, and care of the young. It therefore becomes advantageous to clarify the molecular mechanism of the broodiness in poultry. The pituitary, as a key component of the HPG system, plays a critical role in chicken reproductive performance. In this study, an analysis of whole pituitary transcriptome differences was performed to explore the molecular mechanism underlying the transition from laying to incubation in chickens. The obtained pituitary RNA sequencing data will help us to further understand the biological processes that regulate these two reproductive behaviours.

The incubation behaviour of poultry is mainly affected by the levels of PRL and steroid hormones in plasma. In 1930s, PRL as a hormone of the anterior pituitary was firstly identified to stimulate incubation behaviour in pigeons and chickens [37, 38]. PRL has been known to play a fundamental role in the initiation and maintenance of incubation behaviour in poultry. As expected, transcriptome sequencing revealed that the *PRL* gene was one of the most active DEG in the pituitary, exhibiting an extremely high mRNA level in BP birds. Although it is well established that a characteristic increase of PRL in plasma is closely correlated with the initiation and maintenance of incubation behaviour in domestic and wild birds, the plasma PRL levels seem to be different between precocial and altricial birds. It was reported that the PRL levels in precocial chickens immediately decreased following the hatching of chicks and can be modified by removal of newly hatched chicks from hens [39]. Interestingly, plasma PRL levels in altricial birds seemed to decline more slowly after hatching, presumably because brooding by the parent is imperative for young chicks to survive to fledging.

In addition to PRL, we observed that the expression levels of other hormone-related genes in pituitary such as *PGR*, *RLN3*, *NR5A1* and *GRP* sharply declined in incubating chickens. Similarly, these genes are involved in the initiation and maintenance of incubation behaviour in chickens. PGR as a member of the nuclear receptor superfamily is a specific steroid hormone receptor. The ligand progesterone, a key steroid hormone produced by granulosa cells, can activate PGR and then together they regulate reproductive cycles [40]. RLN3 is the most recently identified member of the relaxin peptide family which may be involved in female reproduction in chickens [41]. It is highly expressed in the pituitary with lower abundance in the hypothalamus, hindbrain, spinal cord and ovary [41]. The expression of *RLN3* is tightly regulated by GnRH, corticotropin-releasing hormone and sex steroid hormone (E2) [41]. The relevant literature suggests that RLN3 might be involved in modulating a range of interrelated functions such as responses to stress, memory, circadian rhythm, sleep/wake states, food intake, metabolism, satiety and neuroendocrine function [42–47]. Meanwhile, preliminary studies have indicated a modulatory role for RLN3 as a pituitary hormone in reproduction and associated behaviour. Transcriptome analysis showed that RLN3 might be related to the transition from laying to brooding in chickens [27, 48]. Both intracerebroventricular and intraparaventricular administration of RLN3 in animals significantly increased the plasma levels of LH, and this effect was blocked by administration of a peripheral GnRH antagonist [49]. GRP is predominantly and abundantly expressed in the anterior pituitary gland and weakly expressed in other tissues in chickens, including the testes, ovary, proventriculus, telencephalon and hypothalamus [50]. In accordance with their wide distribution, GRP is reported to be involved in many physiological processes in poultry, such as circadian clock, food intake, itch sensation, proventriculus acid secretion, gallbladder motility, crop-emptying rate, pancreatic secretion [50–57]. Additionally, evidence has been accumulating to support the notion that pituitary GRP as a novel hormone in chickens may work together with GRP from other tissues to act in a coordinated fashion to regulate reproduction [50]. NR5A1 is an enigmatic orphan receptor essential for the sexual development and reproduction in mammals [58, 59]. Furthermore, it is known to be involved in the expression of pituitary gonadotropic hormone LH and follicle-stimulating hormone (FSH) and the synthesis of progesterone, androgens, estrogens and steroidogenesis [60].

Steroid biosynthesis plays an important role in the transition from the laying to incubation phases in chickens. It usually decreases sharply during the transition from laying to incubation in poultry. Consistently, the steroid biosynthesis pathway was observed to show the most significant changes during the transition from laying to incubation. In this study, eleven DEGs involved in steroid biosynthesis, including *DHCR7*, *LIPA*, *DHCR24*, *SQLE*, *FDFT1*, *HSD17B7*, *LSS*, *CYP51A1*, *SC5D*, *NSDHL* and *MSMO1*, are observed to decrease sharply during the transition from laying to incubation. Furthermore, the neuroactive ligand-receptor interaction pathway was reported to be important for hormonal regulation in reproductive cycle transitions of duck. Similarly, we found that the expression of DEGs associated with the neuroactive ligand-receptor interaction pathway were observed to be significantly different between LP and BP groups, indicating that neuroactive ligand-receptor interaction pathway might play an important role in reproductive cycle transitions of poultry. In this study, the DEGs involved in the TGF-β signaling pathway, including *BMP5*, *BMP2*, *BMP4*, *INHBA*, *INHBB*, *GREM2*, *DCN*, *LTBP1*, *FMOD*, *ADAMTSL2*, *SMURF1* and *SMAD6*, were observed to decrease during the transition from laying to incubation. BMP5, a key member of the TGF-β signaling pathway, is reported to participate in fecundity of animals [61]. Meanwhile, BMP5 could affect ovary development by TGF-β signaling pathway activation [62]. BMP2 has been identified as a candidate gene within the hypothalamic-pituitary-gonadal axis responsible for different egg production performance of ducks [63]. It might be involved in the egg-laying performance of poultry via regulating follicle development and ovulation [63]. In addition, the MAPK pathways in the HPG axis are known to be an intracellular serine/threonine kinase signal transduction pathway that is widely distributed in various tissues including hypothalamus, pituitary glands, and ovaries and can be activated by a variety of stimuli. In chicks, there seem to be gender differences in MAPK pathways in HPG axis [64]. It was reported that heat stress can trigger MAPK crosstalk to turn on the hyperosmotic response pathway [65]. During the early brooding stage, high temperature training was thought to aid chicks in surviving warm environments [64].

In conclusion, the pituitary transcriptome in laying and incubating Changshun hens was evaluated in the present study. We identified a total of 2089 DEGs and found that steroid biosynthesis pathway and neuroactive ligand-receptor interaction were significantly enriched. Five candidate genes, including *SRC*, *ITGB4*, *ITGB3*, *PIK3R3* and *DRD2*, may play crucial roles in the regulation of incubation behaviour in chickens.

### Electronic supplementary material

Below is the link to the electronic supplementary material.

Supplementary Material 1

Supplementary Material 2

Supplementary Material 3

Supplementary Material 4

## Data Availability

The raw sequence data reported in this paper have been deposited in the Genome Sequence Archive (Genomics, Proteomics & Bioinformatics 2021) in National Genomics Data Center (Nucleic Acids Res 2022), China National Center for Bioinformation / Beijing Institute of Genomics, Chinese Academy of Sciences (GSA: CRA012981) that are publicly accessible at https://ngdc.cncb.ac.cn/gsa.

## Abbreviations

HPG: Hypothalamic-pituitary-gonadal
LP: Egg-laying phase
BP: Incubation phase
DEG: Differently expressed gene
GO: Gene Ontology
KEGG: Kyoto Encyclopedia of Genes and Genomes
PPI: Protein-protein interaction
GnRH: Gonadotropin-releasing hormone
VIP: Vasoactive intestinal polypeptide
LH: Luteinizing hormone
DA: Dopamine
5-HTP: 5-hydroxytryptophan
QTL: Quantitative trait loci
FSH: Follicle-stimulating hormone
PRL: Prolactin
RNA-Seq: RNA sequencing
OXT: Oxytocin-neurophysin
CHRDL1: Chordin-like protein 1
GH: Growth hormone
PCA: Principal component analysis
qRT-PCR: Quantitative real-time PCR
CC: Cellular component
MF: Molecular function

## References

[1] Romanov MN, Talbot RT, Wilson PW, Sharp PJ (2002). Genetic control of incubation behavior in the domestic hen. Poult Sci.

[2] El Halawani ME, Burke WH, Millam JR, Fehrer SC, Hargis BM (1984). Regulation of prolactin and its role in gallinaceous bird reproduction. J Exp Zool.

[3] Sharp PJ, Scanes CG, Williams JB, Harvey S, Chadwick A (1979). Variations in concentrations of prolactin, luteinizing hormone, growth hormone and progesterone in the plasma of broody bantams (*Gallus Domesticus*). J Endocrinol.

[4] Sharp PJ, Macnamee MC, Sterling RJ, Lea RW, Pedersen HC (1988). Relationships between prolactin, LH and broody behaviour in bantam hens. J Endocrinol.

[5] Zadworny D, Shimada K, Ishida H, Sumi C, Sato K (1988). Changes in plasma levels of prolactin and estradiol, nutrient intake, and time spent nesting during the incubation phase of broodiness in the Chabo hen (Japanese bantam). Gen Comp Endocrinol.

[6] March JB, Sharp PJ, Wilson PW, Sang HM (1994). Effect of active immunization against recombinant-derived chicken prolactin fusion protein on the onset of broodiness and photoinduced egg laying in bantam hens. J Reprod Fertil.

[7] Sharp PJ, Sterling RJ, Talbot RT, Huskisson NS (1989). The role of hypothalamic vasoactive intestinal polypeptide in the maintenance of prolactin secretion in incubating bantam hens: observations using passive immunization, radioimmunoassay and immunohistochemistry. J Endocrinol.

[8] Richard-Yris MA, Sharp PJ, Wauters AM, Guémené D, Richard JP, Forasté M (1998). Inffuence of stimuli from chicks on behavior and concentrations of plasma prolactin and luteinizing hormone in incubating hens. Horm Behav.

[9] Opel H, Proudman JA (1989). Plasma prolactin levels in incubating Turkey hens during pipping of the eggs and after introduction of poults into the nest. Biol Reprod.

[10] Lea RW, Richard-Yris MA, Sharp PJ (1996). The effect of ovariectomy on concentrations of plasma prolactin and LH and parental behavior in the domestic fowl. Gen Comp Endocrinol.

[11] Macnamee MC, Sharp PJ, Lea RW, Sterling RJ, Harvey S (1986). Evidence that vasoactive intestinal polypeptide is a physiological prolactin-releasing factor in the bantam hen. Gen Comp Endocrinol.

[12] Opel H, Proudman JA (1988). Stimulation of prolactin release in turkeys by vasoactive intestinal peptide. Proc Soc Exp Biol Med.

[13] El Halawani ME, Silsby JL, Mauro LJ (1990). Vasoactive intestinal peptide is a hypothalamic prolactin-releasing neuropeptide in the Turkey (Meleagris gallopavo). Gen Comp Endocrinol.

[14] Rozenboim I, Silsby JL, Tabibzadeh C, Pitts GR, Youngren OM, el Halawani ME (1993). Hypothalamic and posterior pituitary content of vasoactive intestinal peptide and gonadotropin-releasing hormones I and II in the Turkey hen. Biol Reprod.

[15] Youngren OM, Pitts GR, Phillips RE, el Halawani ME (1995). The stimulatory and inhibitory effects of dopamine on prolactin secretion in the Turkey. Gen Comp Endocrinol.

[16] Youngren OM, Pitts GR, Phillips RE, el Halawani ME (1996). Dopaminergic control of prolactin secretion in the Turkey. Gen Comp Endocrinol.

[17] el Halawani ME, Youngren OM, Rozenboim I, Pitts GR, Silsby JL, Phillips RE (1995). Serotonergic stimulation of prolactin secretion is inhibited by vasoactive intestinal peptide immunoneutralization in the Turkey. Gen Comp Endocrinol.

[18] Youngren OM, Silsby JL, Phillips RE, El Halawani ME (1993). Dynorphin modulates prolactin secretion in the Turkey. Gen Comp Endocrinol.

[19] Punnett RC, Bailey PG (1920). Genetic studies in poultry: II. Inheritance of egg-colour and broodiness. J Genet.

[20] Hays FA (1940). Inheritance of broodiness in Rhode Island Reds. Mass Agr Exp Sta Bull.

[21] Saeki Y (1957). Inheritance of broodiness in Japanese Nagoya fowl, with special reference to sex-linkage and notice in breeding practice. Poult Sci.

[22] Saeki Y, Inoue Y (1979). Body growth, egg production, broodiness, age at ffrst age and egg size in red jungle fowls, and attempt at their genetic analyses by reciprocal crossing with White Leghorns. Jpn Poult Sci.

[23] Basheer A, Haley CS, Law A, Windsor D, Morrice D, Talbot R (2015). Genetic loci inherited from hens lacking maternal behaviour both inhibit and paradoxically promote this behaviour. Genet Sel Evol.

[24] Tang J, Liu J, Miao X, Li H, Han X, Li L (2021). Comparative study on Nutritional Components in Eggs of Yaoshan Chicken and Changshun Green-Shell Chicken. J Sichuan Agricultural Univ.

[25] Liu H, Wang J, Li L, Han C, He H, Xu H (2018). Transcriptome analysis revealed the possible regulatory pathways initiating female geese broodiness within the hypothalamic-pituitary-gonadal axis. PLoS ONE.

[26] Ye P, Li M, Liao W, Ge K, Jin S, Zhang C (2019). Hypothalamic transcriptome analysis reveals the neuroendocrine mechanisms in controling broodiness of muscovy duck (Cairina moschata). PLoS ONE.

[27] Ye P, Ge K, Li M, Yang L, Jin S, Zhang C (2019). Egg-laying and brooding stage-specific hormonal response and transcritptional regulation in pituitary of muscovy duck (Cairina moschata). Poult Sci.

[28] Mishra SK, Chen B, Zhu Q, Xu Z, Ning C, Yin H (2020). Transcriptome analysis reveals differentially expressed genes associated with high rates of egg production in chicken hypothalamic-pituitary-ovarian axis. Sci Rep.

[29] Wang C, Ma W (2019). Hypothalamic and pituitary transcriptome profiling using RNA-sequencing in high-yielding and low-yielding laying hens. Sci Rep.

[30] Kim D, Langmead B, Salzberg SL (2015). HISAT: a fast spliced aligner with low memory requirements. Nat Methods.

[31] Pertea M, Pertea GM, Antonescu CM, Chang TC, Mendell JT, Salzberg SL (2015). StringTie enables improved reconstruction of a transcriptome from RNA-seq reads. Nat Biotechnol.

[32] Love MI, Huber W, Anders S (2014). Moderated estimation of Fold change and dispersion for RNA-seq data with DESeq2. Genome Biol.

[33] Young MD, Wakefield MJ, Smyth GK, Oshlack A (2010). Gene ontology analysis for RNA-seq: accounting for selection bias. Genome Biol.

[34] Mao X, Cai T, Olyarchuk JG, Wei L (2005). Automated genome annotation and pathway identification using the KEGG Orthology (KO) as a controlled vocabulary. Bioinformatics.

[35] Shannon P, Markiel A, Ozier O, Baliga NS, Wang JT, Ramage D (2003). Cytoscape: a software environment for integrated models of biomolecular interaction networks. Genome Res.

[36] Livak KJ, Schmittgen TD (2001). Analysis of relative gene expression data using real-time quantitative PCR and the 2– ∆∆CT method. Methods.

[37] Riddle O, Bates RW, Lahr EL (1935). Prolactin induces broodiness in fowl. Am J Physiol.

[38] Riddle O, Bates RW, Dykshorn SW (1933). The preparation, identification and assay of prolactin—a hormone of the anterior pituitary. Am J Physiol.

[39] Kuwayama T, Shimada K, Saito N, Ohkubo T, Sato K, Wada M (1992). Effects of removal of chicks from hens on concentrations of prolactin, luteinizing hormone and oestradiol in plasma of brooding Gifujidori hens. J Reprod Fertil.

[40] Porter TE, Hargis BM, Silsby JL, el Halawani ME (1989). Enhanced progesterone and testosterone secretion and depressed estradiol secretion *in vitro* from small white follicle cells of incubating Turkey hens. Gen Comp Endocrinol.

[41] Lv C, Zheng H, Jiang B, Ren Q, Zhang J, Zhang X (2022). Characterization of relaxin 3 and its receptors in chicken: evidence for relaxin 3 acting as a novel pituitary hormone. Front Physiol.

[42] Smith CM, Ryan PJ, Hosken IT, Ma S, Gundlach AL (2011). Relaxin-3 systems in the brain–the first 10 years. J Chem Neuroanat.

[43] Watanabe Y, Miyamoto Y, Matsuda T, Tanaka M (2010). Relaxin-3/INSL7 regulates the stress-response system in the rat hypothalamus. J Mol Neurosci.

[44] McGowan BM, Stanley SA, Ghatei MA, Bloom SR (2009). Relaxin-3 and its role in neuroendocrine function. Ann N Y Acad Sci.

[45] Higgins SE, Ellestad LE, Trakooljul N, McCarthy F, Saliba J, Cogburn LA (2010). Transcriptional and pathway analysis in the hypothalamus of newly hatched chicks during fasting and delayed feeding. BMC Genomics.

[46] McGowan BM, Stanley SA, Smith KL, White NE, Connolly MM, Thompson EL (2005). Central relaxin-3 administration cuasese hyperphagia in male Wistar rats. Endocrinology.

[47] McGowan BM, Stanley SA, Smith KL, Minnion JS, Donovan J, Thompson EL (2006). Effects of acute and chronic relaxin-3 on food intake and energy expenditure in rats. Regul Pept.

[48] Shen X, Bai X, Xu J, Zhou M, Xu H, Nie Q (2016). Transcriptome sequencing reveals genetic mechanisms underlying the transition between the laying and brooding phases and gene expression changes associated with divergent reproductive phenotypes in chickens. Mol Biol Rep.

[49] McGowan BM, Stanley SA, Donovan J, Thompson EL, Patterson M, Semjonous NM (2008). Relaxin-3 stimulates the hypothalamic-pituitary-gonadal axis. Am J Physiol Endocrinol Metab.

[50] Mo C, Huang L, Cui L, Lv C, Lin D, Song L (2017). Characterization of NMB, GRP and their receptors (BRS3, NMBR and GRPR) in chickens. J Mol Endocrinol.

[51] Tachibana T, Matsuda K, Khan SI, Ueda H, Cline MA (2010). Feeding and drinking response following central administrations of bombesin-like peptides in chicks. Comp Biochem Physiol Mol Integr Physiol.

[52] Tachibana T, Matsuda K, Sawa H, Mikami A, Ueda H, Cline MA (2010). Differential thresholds of neuromedins B-, C-, and bombesin-induced anorexia and crop-emptying rate in chicks. Gen Comp Endocrinol.

[53] Sun YG, Chen ZF (2007). A gastrin-releasing peptide receptor mediates the itch sensation in the spinal cord. Nature.

[54] Sun YG, Zhao ZQ, Meng XL, Yin J, Liu XY, Chen ZF (2009). Cellular basis of itch sensation. Science.

[55] Campbell BJ, Garner A, Dockray GJ, Hughes J, Dimaline R (1994). The mechanism of action of gastrin releasing peptide (GRP) in stimulating avian gastric acid secretion. Regul Pept.

[56] Linari G, Linari MB (1975). Effect of bombesin on pancreatic secretion and gall bladder motility of the chicken. Eur J Pharmacol.

[57] Kallingal GJ, Mintz EM (2007). Gastrin releasing peptide and neuropeptide Y exert opposing actions on circadian phase. Neurosci Lett.

[58] Mallet D, Bretones P, Michel-Calemard L, Dijoud F, David M, Morel Y (2004). Gonadal dysgenesis without adrenal insufficiency in a 46, XY patient heterozygous for the nonsense C16X mutation: a case of SF1 haploinsufficiency. J Clin Endocrinol Metab.

[59] Hasegawa T, Fukami M, Sato N, Katsumata N, Sasaki G, Fukutani K (2004). Testicular dysgenesis without adrenal insufficiency in a 46,XY patient with a heterozygous inactive mutation of steroidogenic factor-1. J Clin Endocrinol Metab.

[60] Jameson JL (2004). Of mice and men: the tale of steroidogenic factor-1. J Clin Endocrinol Metab.

[61] Li X, Ye J, Han X, Qiao R, Li X, Lv G (2020). Whole-genome sequencing identifies potential candidate genes for reproductive traits in pigs. Genomics.

[62] Divya D, Bhattacharya TK (2021). Bone morphogenetic proteins (BMPs) and their role in poultry. Worlds Poult Sci J.

[63] Yan X, Liu H, Hu J, Han X, Qi J, Ouyang Q (2022). Transcriptomic analyses of the HPG axis-related tissues reveals potential candidate genes and regulatory pathways associated with egg production in ducks. BMC Genomics.

[64] Li QH, Yu ZQ, Chen Z (2020). Effect of heat stress on mitogen-activated protein kinases in the hypothalamic-pituitary-gonadal axis of developing Wenchang chicks. Poult Sci.

[65] Dunayevich P, Baltanás R, Clemente JA, Couto A, Sapochnik D, Vasen G (2018). Heat-stress triggers MAPK crosstalk to turn on the hyperosmotic response pathway. Sci Rep.

