# Supplementary material for: Pituitary transcriptome profile from laying period to incubation period of Changshun green-shell laying hens
Source: BMC Genomics. 2024 Mar 25;25:309. doi: 10.1186/s12864-024-10233-1 (PMC10962202; doi:10.1186/s12864-024-10233-1)
Supplement: Supplementary file 1 — Supplementary Material 1 [file 12864_2024_10233_MOESM1_ESM.docx]

**Table S1**. Primers used for qRT-PCR.

| **Gene Name** | **Primer Sequence (5’-3’)** |
| --- | --- |
| *DHCR7* | FP: AGGACTGATAGCCGGGCA  RP: ACCAGTCTACCTCCCATGCT |
| *SC5D* | FP: CTTAGAGAACCAGGTGCAACG  RP: TACAGTTTGCTGTAGCCCCG |
| *PRL* | FP: AAAGCTGTTAATGGCTGCCAC  RP: TCATTCCAGGAACGCAGCAC |
| *BMP5* | FP: AACCGCAACAAATCCAGTAGTC  RP: AGTCCTGCCATCCCAAATCTCG |
| *β-actin* | FP: GAGAAATTGTGCGTGACATGA  RP: CCTGAACCTCTCATTGCCA |
