# Supplementary material for: Pituitary transcriptome profile from laying period to incubation period of Changshun green-shell laying hens
Source: BMC Genomics. 2024 Mar 25;25:309. doi: 10.1186/s12864-024-10233-1 (PMC10962202; doi:10.1186/s12864-024-10233-1)
Supplement: Supplementary file 2 — Supplementary Material 2 [file 12864_2024_10233_MOESM2_ESM.docx]

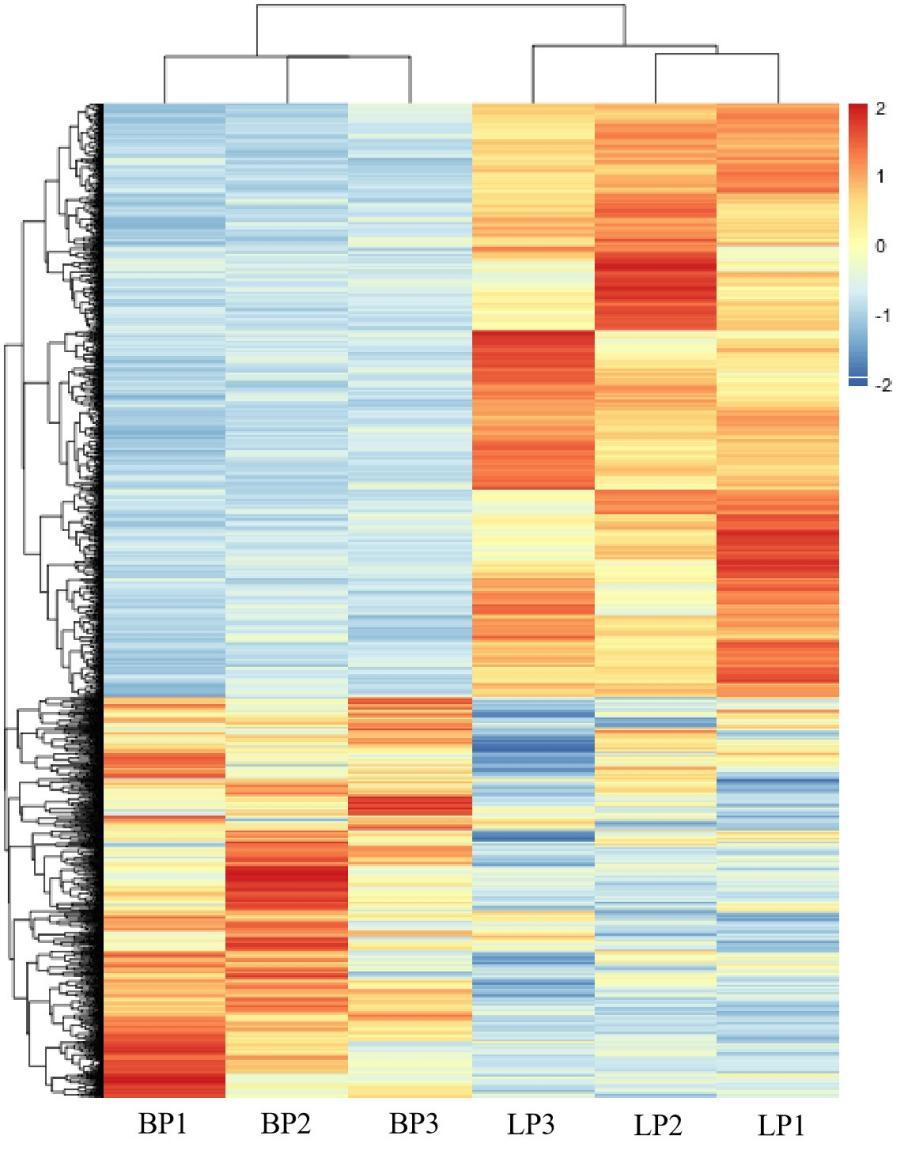


**Figure S1**. Hierarchical clustering analysis of DEGs. LP and BP represent pituitary samples of egg-laying phase hens and incubation phase hens, respectively. The red blocks represent the overexpressed genes, and the blue blocks represent genes with the lowest expression levels. Colored bars indicate the expression levels.
