# Supplementary material for: Pituitary transcriptome profile from laying period to incubation period of Changshun green-shell laying hens
Source: BMC Genomics. 2024 Mar 25;25:309. doi: 10.1186/s12864-024-10233-1 (PMC10962202; doi:10.1186/s12864-024-10233-1)
Supplement: Supplementary file 3 — Supplementary Material 3 [file 12864_2024_10233_MOESM3_ESM.docx]

**Table S3**. List of GO enrichment analysis of DEGs.

| GO category | GO subcategory | Number of DEGs |
| --- | --- | --- |
| cellular component | intracellular | 747 |
|  | protein-containing complex | 282 |
|  | other organism part | 1 |
|  | cellular anatomical entity | 1269 |
| molecular function | catalytic activity | 526 |
|  | structural molecule activity | 48 |
|  | transporter activity | 141 |
|  | binding | 1067 |
|  | antioxidant activity | 7 |
|  | cargo receptor activity | 14 |
|  | translation regulator activity | 9 |
|  | molecular transducer activity | 149 |
|  | molecular function regulator | 153 |
|  | transcription regulator activity | 133 |
| biological process | reproduction | 175 |
|  | immune system process | 133 |
|  | behavior | 94 |
|  | metabolic process | 600 |
|  | cellular process | 1149 |
|  | reproductive process | 175 |
|  | biological adhesion | 105 |
|  | signaling | 473 |
|  | multicellular organismal process | 508 |
|  | developmental process | 481 |
|  | growth | 63 |
|  | locomotion | 187 |
|  | pigmentation | 9 |
|  | interspecies interaction between organisms | 81 |
|  | rhythmic process | 26 |
|  | response to stimulus | 621 |
|  | localization | 434 |
|  | intraspecies interaction between organisms | 8 |
|  | multi-organism process | 162 |
|  | biological regulation | 900 |
|  | detoxification | 7 |
|  | biomineralization | 6 |
